# A Rare Case of Enterococcus gallinarum-Associated Peritonitis and Literature Review

**DOI:** 10.7759/cureus.12328

**Published:** 2020-12-27

**Authors:** Paul Nguyen, Suman Khicher, Heba Osman, Neel Patel

**Affiliations:** 1 Internal Medicine, Wayne State University School of Medicine, Detroit, USA; 2 Rheumatology, Wayne State University School of Medicine, Detroit, USA

**Keywords:** peritonitis, peritoneal dialysis, vancomycin resistant enterococcus (vre), vancomycin, daptomycin, multi-drug resistant organism (mdro)

## Abstract

Peritonitis is a well-known complication seen with peritoneal dialysis. Peritonitis is associated with increased mortality risk and is commonly caused by gram-positive and gram-negative bacteria, but it can also be the result of fungal or viral infections. Therefore, it is imperative to obtain a peritoneal fluid sample to send for cell count with differential, gram stain, and culture prior to starting empiric antibiotic therapy. We report a case of peritoneal dialysis-related peritonitis caused by *Enterococcus gallinarum,* for which there has only been one other reported case in the medical literature. Our patient was initially placed on vancomycin and cefepime but continued to deteriorate until peritoneal fluid cultures revealed *E. gallinarum.* Based on sensitivities, the patient was treated with daptomycin and cefazolin, which resolved her peritonitis.

## Introduction

Peritonitis is a well-known complication seen with peritoneal dialysis (PD). The bacteria most commonly seen are gram-positive and gram-negative organisms. Another gram-positive organism that is seen in peritonitis is the *Enterococcus* species. *Enterococci* are facultative anaerobes found in the normal human gut flora and can lead to infections from translocation and contact contamination. Vancomycin-resistant enterococci (VRE) have been described since the early 1980s and since then has posed therapeutic challenges for physicians. These challenges have warranted antibiotics combinations to achieve synergy for eradicating resistant *Enterococcal *infections, i.e., infectious endocarditis and peritonitis. 

## Case presentation

An 85-year-old female patient with a history of coronary artery disease, aortic valve replacement (AVR), type 2 diabetes mellitus, and end-stage renal disease (ESRD) on PD presented to the emergency department with difficulty breathing and altered mental status. Her son, the primary caregiver, shared that the patient was recently discharged from subacute rehab following a below-knee amputation (BKA) and had been having diarrhea. No further review of systems was obtainable due to her clinical status.

Upon arrival, her blood pressure was 113/48 mmHg, heart rate of 69 beats per minute, respiratory rate of 20 breaths per minute, oxygen saturation of 99% on 100% non-rebreather mask, the temperature of 36.6° C. Physical exam revealed an elderly, frail female who was not oriented. She had bilateral coarse rhonchi on auscultation of her lungs with decreased breath sounds at the right lung base. The heart exam was normal. The abdomen was tender to palpation as facial grimacing was noted. A PD catheter was in place, and the site was clean. The patient did not follow commands, but the neurologic exam was non-focal. 

**Figure 1:**
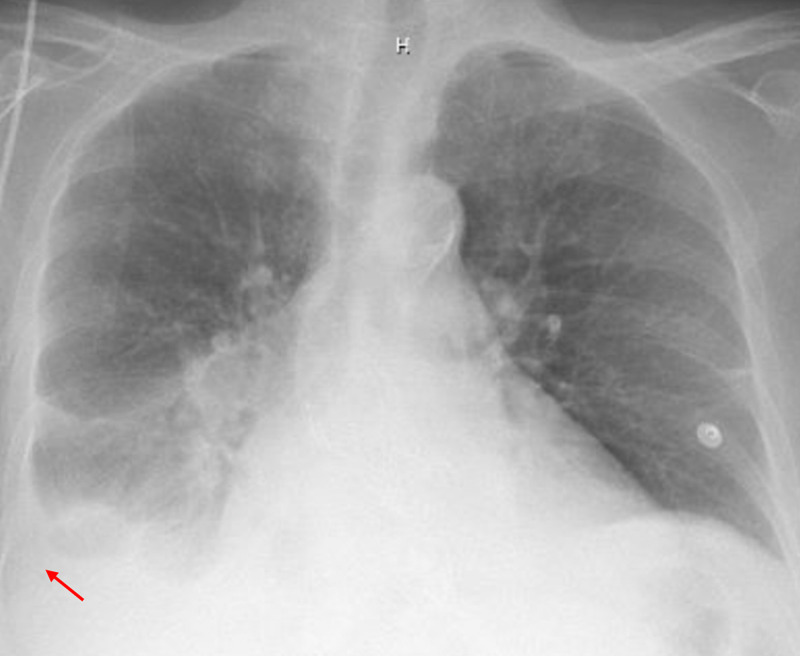
Chest X-ray depicting right-sided pleural effusion (red arrow)

Further workup revealed blood urea nitrogen (BUN) 27 mg/dL, creatinine 3.8 mg/dL (at baseline), mild hypokalemia at 3.5 mMol/L, leukocytosis at 13.5/mm^3^. A stat computed tomography of her head showed no acute intracranial process, and a chest X-ray revealed a right pleural effusion with possible right lower lobe opacities, which were unchanged from the previous studies (Figure 1). 

She was then started on empiric antibiotics, including vancomycin, cefepime, and doxycycline, and admitted to the intensive care unit for severe sepsis. Infectious disease was consulted and recommended switching cefepime to ceftriaxone as the patient’s mental status continued to deteriorate, in addition to sending peritoneal fluid for analysis and culture. While awaiting PD fluid cultures, her blood cultures grew Methicillin-resistant *Staphylococcus aureus *(MRSA), leading to concerns for infective endocarditis given her AVR history. The peritoneal fluid analysis revealed neutrophilic predominance with results as follows: 3,938 nucleated cells, 82% neutrophils, 9% lymphocytes, 8% monocytes, and 1% eosinophils. Although receiving empiric antibiotic therapy, the patient’s clinical condition continued to deteriorate. On the fifth hospital day, the patient’s peritoneal fluid culture revealed *Enterococcus gallinarum *resistant to vancomycin. The patient was subsequently placed on IV daptomycin and cefazolin for synergy with improvement in her mental status within 48 hours. Repeat peritoneal fluid analysis showed decreased leukocytes and clearance of her *E. gallinarum* on repeated fluid cultures. The susceptibilities for *E. gallinarum *are listed in Table 1.

**Table 1:** Susceptibilities for Enterococcus gallinarum MIC - minimum inhibitory concentration; ET2SUSP - electrophoretic type 2 susceptibility; ET2 - electrophoretic type 2; S - susceptible; Gent Syn - sentamycin synergy

	MIC	ET2SUSP	Interpretation	ET2 interpretation
Antimicrobial	Rare Enterococcous gallinarum	Rare Enterococcous gallinarum		
Ampicillin	1		S	
Daptomycin		4		S
Gent Syn Screen	≤ 500		S	
Linezolid		2		S
Streptomycin synergy	≤ 1000		S	

**Figure 2:**
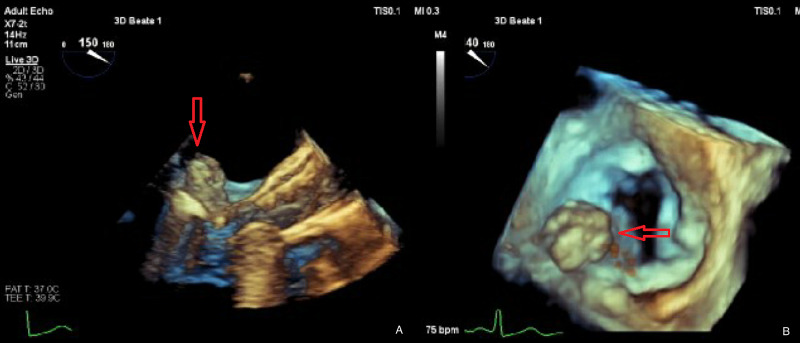
A: Midesophageal long-axis view showing large abscess of MV (red arrow). B: Atrial view of the MV showing abscess of posterior leaflet (red arrow). MV - mitral valve

**Figure 3:**
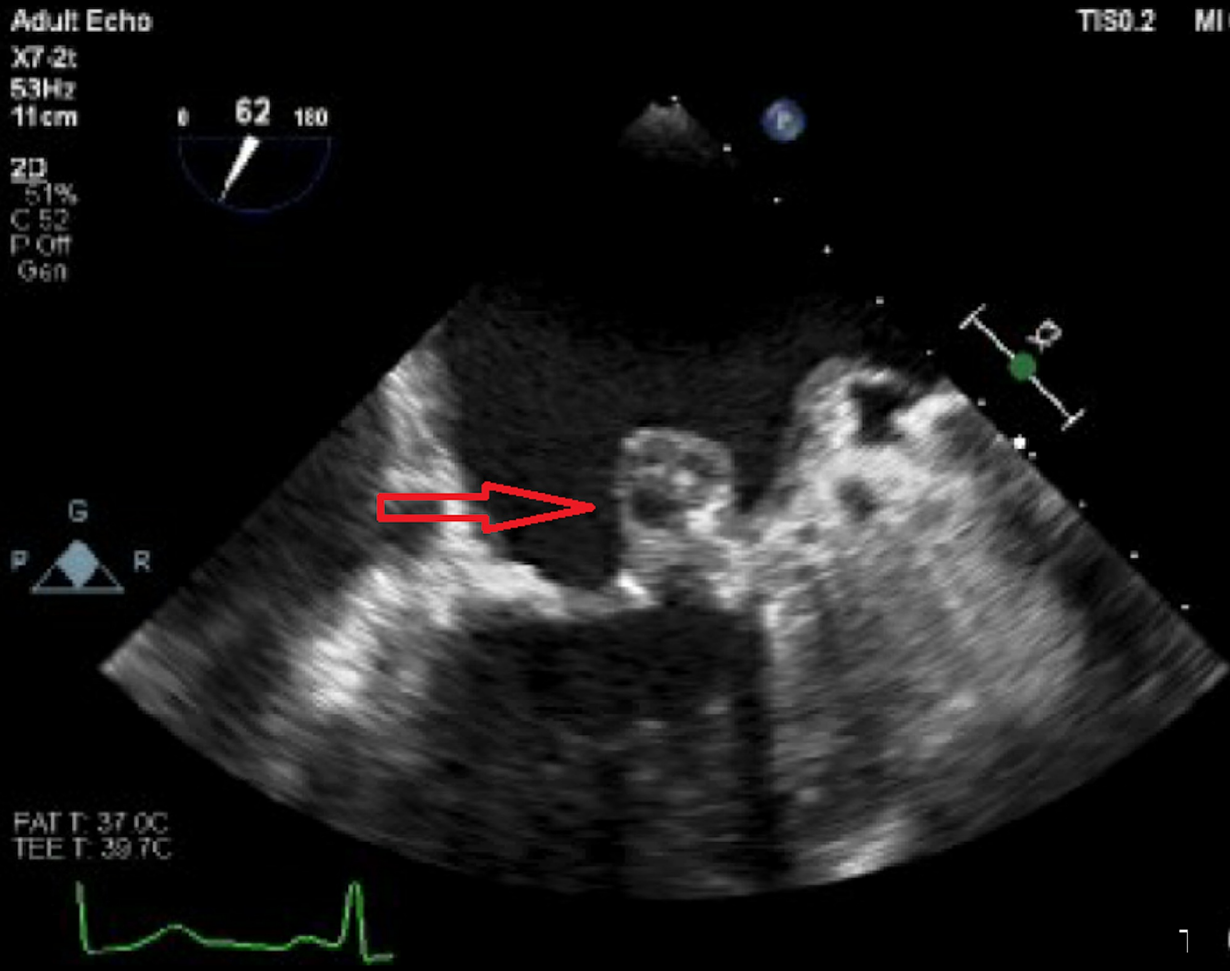
Two-chamber view showing abscess of mitral leaflet (red arrow)

A transthoracic echocardiogram was performed as the patient also had persistent MRSA bacteremia, which did not show any vegetations or mass. A transesophageal echocardiogram (TEE) subsequently revealed a large globular deformation of the posterior mitral leaflet bulging into the left atrium, measuring 2.0 x 1.9 cm in size that was concerning for mitral valve abscess (Figures 2-3). Unfortunately, due to the patient's worsening clinical status and considering the overall prognosis, the patient was placed in hospice care and died several days later. An autopsy was not performed. 

## Discussion

PD-related peritonitis is a widely known serious complication of PD as it is associated with increased mortality. It is also the leading cause of PD failure requiring transitioning to hemodialysis (HD) [1]. The most common gram-positive organisms seen are *Staphylococcus aureus* and other *Staphylococcus spp.* along with *Escherichia coli *and *Klebsiella sp.* for gram-negative organisms [2, 3]. Although VRE is not commonly seen in PD-related peritonitis, it alone increases the transition to permanent HD [4]. *Enterococcus *species are an essential component of the intestinal microbiota of healthy humans and animals [5, 6]. However, they can also cause opportunistic infections, which have been documented, leading to millions of annual infections [7]. There have been only a few reported cases of *E. gallinarum* peritonitis (Table 2); all have been associated with cirrhosis, i.e., part of spontaneous bacterial peritonitis, not PD-related peritonitis [8-12]. We present the second reported case in the medical literature of PD-related peritonitis caused by *E. gallinarum* [13]. It is not clear how our patient contracted *E. gallinarum* peritonitis, but it is likely due to fecal contamination. *E. gallinarum* has been reported as a rare cause of infective endocarditis (IE) in literature [14, 15]. Post-mortem evaluation of the valve tissue remains the gold standard for IE diagnosis as blood culture does not detect organisms in about one-third of the cases [16], so there can be a possibility of polymicrobial IE due to both MRSA and VRE in our patient.

**Table 2:** Reported cases of spontaneous bacterial peritonitis caused by Enterococcus gallinarum HCV - hepatitis C virus

Reference	Age/Sex	Cause of cirrhosis	Absolute neutrophil count	Antibiotic used	Outcome
Abidali et al. 2015 [8]	60/Male	HCV	944 cells/𝜇L	Ampicillin/Sulbactam	Survived
Alvarez et al. 2002 [9]	67/Female	HCV	Normal	Ampicillin	Survived
Narisco-Schiavon et al. 2015 [10]	63/Female	Non-alcoholic steatohepatitis	2,610/mm^3^	Ampicillin & Gentamicin	Survived
Redondo-Cerezo et al. 2002 [11]	74/Female	HCV	810/mL	Ampicillin	Expired
Reuken et al. 2012 [12]	-	-	-	-	-

*E. gallinarum* is one of the species in the *Enterococcus* family that is resistant to vancomycin. Vancomycin resistance is categorized as either low or high-level resistance. Vancomycin binds to the D-alanyl-D-alanine (D-Ala-D-Ala) moieties and prohibits cell wall synthesis. *Enterococci* develop resistance by substituting D-Ala-D-Ala for D-Alanine-D-Lactate (D-Ala-D-Lac) or D- Alanine-D-Serine (D-Ala-D-Ser) [17, 18]. The vanC operon transcribes a D-Serine and replaces the end terminus of D-Ala. This allows *E. gallinarum *to demonstrate low-level resistance (minimum inhibitory concentration [MIC], 4 to 32 μg/mL) [19, 20]. *Enterococci* can develop resistance to our last resort antimicrobials used to treat glycopeptide and multidrug resistance (such as quinupristin-dalfopristin, linezolid, daptomycin, and tigecycline) [19]. Moreover, prior exposure to vancomycin adds a risk for drug resistance. Thus, once one obtains a culture for *E. gallinarum, *it is incumbent on the clinician to modify therapy as all *E. gallinarum* are resistant to vancomycin. One does not need to wait for sensitivities. However, if dealing with a concomitant MRSA infection with VRE providers can start daptomycin or linezolid.

## Conclusions

In this case report, we report the second known case of PD-related peritonitis caused by *E. gallinarum*. Although, the most common causes of PD-related peritonitis are *Staphylococcus aureus* and other *Staphylococcus spp.* along with *Escherichia coli *and *Klebsiella sp.*; clinicians should be cognizant of other multidrug-resistant organisms in patients who do not respond to typical broad-spectrum antibiotics as antibiotic resistance continues to persist.
